# Fostering eating after stroke (FEASt) trial for improving post-stroke dysphagia with non-invasive brain stimulation

**DOI:** 10.1038/s41598-022-14390-9

**Published:** 2022-06-10

**Authors:** Sandeep Kumar, Sarah Marchina, Susan Langmore, Joseph Massaro, Joseph Palmisano, Na Wang, David Eric Searls, Vasileios Lioutas, Jessica Pisegna, Cynthia Wagner, Anant Shinde, Gottfried Schlaug

**Affiliations:** 1 Department of Neurology, Stroke Division, Beth Israel Deaconess Medical Center, 330 Brookline Ave, Palmer 127, Boston, MA 02215 USA; 2 Harvard Medical School, Boston, MA USA; 3 Department of Otolaryngology, Boston University School of Medicine, Boston, MA USA; 4 Biostatistics, Boston University School of Public Health, Boston, MA USA; 5 Biostatistics and Epidemiology Data Analytics Center (BEDAC), Boston University School of Public Health, Boston, MA USA; 6 Department of Neurology, UMass Chan Medical School-Baystate, Springfield, MA USA; 7 Department of Biomedical Engineering/Institute of Applied Life Sciences, UMass Amherst, Amherst, MA USA

**Keywords:** Neuroscience, Synaptic plasticity

## Abstract

Dysphagia is a serious stroke complication but lacks effective therapy**.** We investigated safety and preliminary efficacy of anodal transcranial direct current stimulation (atDCS) paired with swallowing exercises in improving post-stroke dysphagia from an acute unilateral hemispheric infarction (UHI). We conducted a double-blind, early phase-2 randomized controlled trial, in subjects (n = 42) with moderate-severe dysphagia [Penetration and Aspiration Scale (PAS) score ≥ 4], from an acute-subacute UHI. Subjects were randomized to Low-Dose, High-Dose atDCS or Sham stimulation for 5 consecutive days. Primary safety outcomes were incidence of seizures, neurological, motor, or swallowing function deterioration. Primary efficacy outcome was a change in PAS scores at day-5 of intervention. Main secondary outcome was dietary improvement at 1-month, assessed by Functional Oral Intake (FOIS) score. No differences in pre-defined safety outcomes or adjusted mean changes in PAS, FOIS scores, between groups, were observed. Post-hoc analysis demonstrated that 22 /24 subjects in the combined atDCS group had a clinically meaningful dietary improvement (FOIS score ≥ 5) compared to 8 /14 in Sham (*p* = 0.037, Fisher-exact). atDCS application in the acute-subacute stroke phase is safe but did not decrease risk of aspiration in this early phase trial. The observed dietary improvement is promising and merits further investigation.

## Introduction

Dysphagia is a very common complication of a stroke and carries major implications for stroke survivors^1–3^. It is independently associated with increased risk of pneumonia and malnutrition, early stroke mortality and institutionalization^1,2,4–6^. Treatments that help restore normal swallowing can therefore improve stroke outcomes. Unfortunately, such therapies are currently lacking and management usually revolves around providing nutritional support with nasogastric or percutaneous endoscopic gastrostomy tubes until swallowing recovers spontaneously, if at all^7^; patients fed via these means remain vulnerable to serious medical complications and have been observed to have high mortality rates^8–10^.

The prevailing view of swallowing and its control has evolved to incorporate the great diversity of swallowing behavior in health that can range from reflex swallowing to volitional eating or a combination of volitional and reflexive swallowing such as brisk drinking. Both cerebral hemispheres, the subcortical regions and the central pattern generators in the medullary pontine regions intricately mediate normal swallowing^10,11^. This improved understanding of swallowing behavior and its regulation aligns well with the observation that patients with isolated unilateral hemispheric strokes that spare the brainstem structures also have a high incidence of persistent dysphagia^2,3,12^.

Recent insights on the neurophysiology of swallowing control in health and the neuroplastic processes that drive swallowing recovery after stroke also provide a basis to develop and test targeted therapies for post-stroke dysphagia. Separate investigations show that while unilateral hemisphere infarction can produce dysphagia by damaging the swallowing motor cortex or its projections, the contralateral healthy swallowing cortex possesses the capacity to reorganize sufficiently to enable restoration of normal swallowing^13–15^. The presence of inherent redundancies in swallowing control, where projections from both swallowing cortices converge on a common pool of brainstem structures (“swallowing-centers”), can enable independent unilateral hemispheric regulation of bilateral “swallowing-centers”^16^. These unique features make the undamaged swallowing cortex an attractive target for cortical modulation techniques to augment the naturally occurring neuroplastic changes within this region and to enhance its role as a mediator of recovery^17,18^.

As hemispheric strokes are the commonest stroke sub-type in the population, they likely contribute disproportionately to the overall dysphagia burden^2,12,19^. A non-invasive brain stimulation technique, like transcranial direct current stimulation (tDCS) may be especially well suited to improve swallowing in this stroke population^16,17^. It has been demonstrated that recovery of swallowing functions in patients with hemispheric lesions occurs via compensatory reorganization of the undamaged cerebral hemisphere and shows a predictable pattern of expansion of the pharyngeal representation in an anterolateral direction, irrespective of lesion site or laterality^15^. This reorganization is preceded by an increase in excitability of the swallowing cortex in the undamaged hemisphere suggesting that the excitability changes drive cortical reorganization^15^. We therefore hypothesize that with hemispheric lesions, where the brainstem and peripheral structures are intact but the upper echelons of the swallowing apparatus are dysfunctional, a cortical stimulation technique can be effective by engaging the intact swallowing motor cortex and helping to restore swallowing functions.

We and others have previously reported on the effect of anodal tDCS (atDCS) for dysphagia recovery after a stroke in pilot studies^20,21^. Building upon these experiences, we systematically examined the safety and feasibility, and performed a preliminary assessment of efficacy of atDCS paired with swallowing exercises in improving swallowing functions after an acute unilateral hemispheric infarction in a randomized controlled clinical trial (RCT).

## Methods

### Standard protocol approvals, registrations, and patient consents

The study was reviewed and approved by the Institutional Review Board at Beth Israel Deaconess Medical Center. We confirm that all research procedures was performed in accordance with relevant regulations and in accordance with the Declaration of Helsinki. A written informed consent was obtained for all trial participants or their legal guardians. A Data Safety Monitoring Board with assistance from a Medical Safety Monitor reviewed the progress of the trial. This clinical trial was registered on Clinicaltrials.gov (URL: http://www.clinicaltrials.gov. Unique identifier: NCT01919112) on 8/08/ 2013.

### Trial design and participants

FEASt is an early phase-2 double-blind, single-center RCT conducted from September 2013–September 2019. The rationale, design and protocol of the trial have been published previously^22^. All subjects were recruited at Beth Israel Deaconess Medical Center (BIDMC) in Boston, MA. We enrolled patients with an acute-subacute unilateral hemispheric infarction, day 2-day 6 since the qualifying stroke, between 21–90 years of age, with moderate to severe dysphagia, defined as a Penetration And Aspiration Scale^23^ (PAS) score ≥ 4 on a standardized videofluoroscopic study (VFSS). The main exclusion criteria were pre-stroke swallowing difficulties, severe stroke (NIHSS score ≥ 25), or presence of any other contraindication for tDCS^22^.

### Randomization and masking

Subjects were randomized to 1 of 3 intervention arms–a Low-Dose tDCS, a High-Dose tDCS, or a Sham group, using computer based randomization stratified according to the baseline PAS scores (4–6 versus 7–8). Randomization codes were generated electronically by an independent study Data Coordination Center (DCC). Three individuals, not involved in any other study procedure received confidential electronic notification about the randomization codes and programmed the tDCS device accordingly. They also interrogated the device after each session to verify its fidelity to the stimulation allocation. All de-identified VFSS were sent electronically to the Boston University Medical Center laboratory for detailed analysis and results were fed directly to the DCC. These processes ensured complete concealment of intervention allocation and blinding of all investigators and subjects.

### Swallowing procedures and outcomes assessments

All consecutive acute ischemic stroke (AIS) patients admitted to BIDMC were screened for trial participation. All patients received a standardized dysphagia screening using a 3-oz water swallow test^24^ and underwent a swallow evaluation by a Speech-Language pathologist (SLP) if suspected of having dysphagia. Those with moderate to severe dysphagia (PAS ≥ 4) on a standardized VFSS evaluation were enrolled if they fulfilled all study criteria. The VFSS protocol employed 3 consistencies (nectar, pudding and thin liquids) and 5 swallows at trial enrollment (baseline) and after the 10th or last stimulation session (post-stimulation). The arithmetic mean of the PAS score was computed for each subject (composite PAS score) at these time points. A Functional Oral Intake Scale (FOIS) score^25^ was computed by the study SLP at baseline, after 5th and final session; in addition, at 30-days after the index stroke, an investigator, blinded to the intervention allocation, collected a FOIS score over the phone or in-person using a standardized questionnaire with the subject or a legally authorized representative familiar with subject’s current dietary status. An investigator certified in the performance of the NIH Stroke Scale (NIHSS)^26^ assigned scores for each participant at enrollment, after every second stimulation sessions and after the last session. All subjects underwent a brain MRI with diffusion weighted imaging (DWI) prior to enrollment, which were used to create a binary map of the acute stroke lesion and construct a novel radiological variable, the corticobulbar tract-lesion load (CBT-LL) (below).

The primary efficacy outcome was a change in PAS scores based on VFSS^22^ from baseline to post-stimulation.. PAS is specifically designed to measure the depth of airway invasion from a swallow bolus (degrees of penetration and aspiration) and the compensatory response to expel this bolus from the airways (effective, ineffective or absent cough)^23^. We had selected PAS scores as a primary measure of efficacy since aspiration has been reported to be a major contributor to pneumonia and dietary restriction after stroke^27^. We supplemented our assessment of intervention efficacy with our secondary outcomes which included objective measures of dietary improvement assessed by a change in FOIS scores 30-days post-stimulation^25^ and changes in swallowing physiology parameters on VFSS from baseline to post-stimulation [pharyngeal delay time (PDT), pharyngeal constriction ratio (PCR), and hyoid, laryngeal and pharyngeal excursion (HLPE)]^22,28–30^. The primary safety outcomes were incidence of seizures, deterioration in global neurological functions (≥ 4-point increase in the total NIHSS score), motor functions (≥ 2 points increase in the motor sub-item of the NIHSS score on the same limb), swallowing functions (increase in PAS score by ≥ 2 points compared to baseline), and stroke specific mortality during the period of active stimulation.

The CBT-LL variable was derived by creating a canonical tract of the corticobulbar tract in spatially standardized space using the swallowing related cortical fMRI activation and the posterior pons as seed regions. The fMRI experiment consisted of repeated swallowing trials contrasted with whole hand opening and closing tasks, done at the same frequency as the swallowing tasks. The fMRI sequences were acquired with a gradient-echo T2*-weighted MR pulse sequence using our own modification of a sparse temporal sampling method with clustered volume acquisition to overcome imaging artifacts caused by swallowing motion. The voxel clusters of significant cortical activation were used as a seed region (to identify the cortical origin of the CBT) with the second seed region in the posterior pons. The DTI scans were high resolution studies obtained in 12 healthy elderly controls. Image acquisition, analysis, construction of corticobulbar tracts and computation of CBT-lesion load were performed as previously described by Zhu et al.^31^. Overlaying the manually drawn lesion maps derived from the acute DWI sequences of trial participants onto this canonical probabilistic CBT allowed us to calculate a CBT-lesion-load variable. This overcomes previous shortcomings in describing lesion volume and lesion location by creating a combined lesion load variable that combines lesion volume and relevant lesion location that overlay a relevant system such as the CBT-lesion load. For clinical interpretability, the CBT-LL variable was dichotomized for each subject based on their lesion-load measures , into 2 groups (< group median, ≥ group median) to assess for effect modification of this variable on the experimental intervention.

We performed a post-hoc analysis using a FOIS score threshold ≥ 5 as clinically meaningful improvement in dietary intake based on a recent noteworthy publication that utilized this threshold to develop an instrument for prognosticating dysphagia recovery^32^. This cut-off corresponds to a dietary intake of a single consistency or worse and demonstrated to correlate with significant reduction in protein, energy and fluid intake, and suggested as an indication for gastrostomy tube placement after stroke^33–35^.

### Experimental intervention

**Figure 1:**
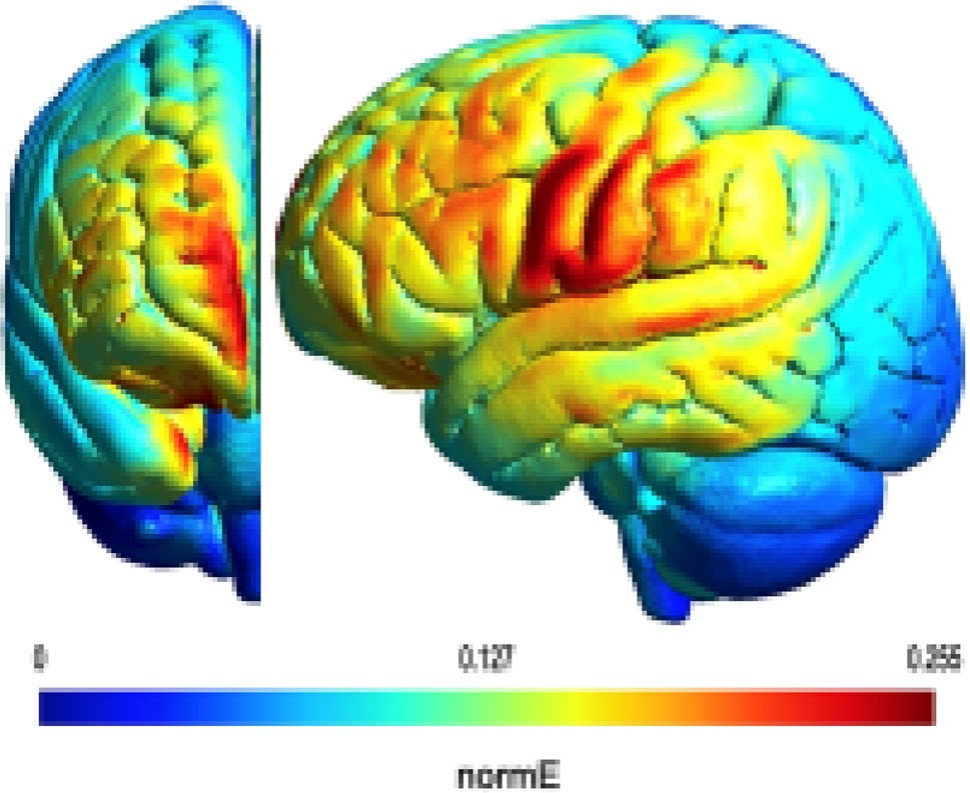
The simulated electric field (normal to the cortical surface) with a rectangular anodal electrode of 3 × 5 cm positioned with its center at the half-way point between C3 and T3 (left) or C4 and T4 (right) on the unaffected hemisphere respectively, and a rectangular cathodal electrode of 5 × 7 cm placed long-way over the surpraorbital region on the affected hemisphere.

The stimulation session were performed for 20 min twice daily over 5 consecutive days. Anodal tDCS (2 mA) or sham was delivered via a battery-driven, constant current stimulator (NeuroConn-DC Stimulator Plus) using saline soaked electrodes (anode 3 × 5 cm; reference electrode 5 × 7 cm). We targeted the healthy swallowing motor cortex using the 10–20 EEG electrode placement system, placing the anode mid-distance between C3/T3 [left] or C4/T4 [right] over the unaffected hemisphere and the reference electrode over the contralateral supraorbital region. We had previously verified the location of the stimulating electrode using a combination of functional brain MRI (fMRI) and anatomical brain MRI scans^21^. The electrode positioning was re-confirmed in a subset of trial participants using anatomical brain MRI scans. The atDCS electric field (V/m) distribution was modeled using a freely available software package called SimNIBS^36^ (Fig. 1). The MNI ICBM 152 1 mm T1 weighted image was used to perform simulation^37^. As shown in Fig. 1, the electric field distribution (V/m) is influenced by the shape, location, and current applied through the electrodes.

The High-Dose tDCS group received 2 mA atDCS twice daily for a total of 20 min (total charge density 16 C/cm2); Low-Dose group received 2 mA alternating with sham stimulation daily for a total of 20 min (total charge density 8 C/cm2) and Sham group received sham stimulation twice daily.

All stimulation sessions were conducted concurrently with standardized effortful swallowing exercises^38^. All study SLPs and investigators were trained by an expert (SL) on eliciting these maneuvers. Occurrence of an effortful swallow was verified by a laryngeal microphone attached to the patient’s throat. We aimed to obtain 40 effortful swallows during each session, which were recorded and entered into the study website.

### Statistical analysis

We planned to randomize 99 subjects- 33 in each arm, estimated to detect a difference of 1.0 and 1.15 standard deviations (SD) between groups in the mean primary outcome measure with a type I error rate of 2.5% and power 80% and 90%, respectively.

The primary efficacy analysis was performed in an intent-to-treat approach. Our outcome variable was a change in the arithmetic mean PAS scores (averaged for 5 different swallows at each time point), before and after intervention and compared across groups. We used a linear model using PROC GLM in SAS to analyze the mean change in PAS scores with the intervention after adjusting for baseline NIHSS scores and age, which were included as covariates based on clinical considerations after screening for other confounders. Similar analyses were performed on the dietary outcome (mean change in FOIS scores at 30-days) and other secondary analysis on changes in swallowing physiology (PDT, PCR, and HLPE). The incidence of safety outcomes were captured as a whole and compared separately across groups. Intraclass correlation (ICC) for PAS scores was performed on a subset of VFSS (randomly selected 303 swallow evaluations) between 2 reviewers (JP and SL). Regression models using interaction terms were used to assess for heterogeneity of intervention effects across the CBT-LL.

In addition, we conducted the following post-hoc analysis: 1) PRESS score, a recently validated prognostic tool for spontaneous swallowing recovery after an AIS, was computed for each participant and incorporated in an adjusted analysis for the efficacy outcomes^39^; 2) the total number of experimental sessions (10 or < 10) for every subject was used as a covariate in an adjusted analysis for our primary outcome; 3) Fisher-exact test was conducted to compare the proportion of subjects with a FOIS score ≥ 5 in the combined atDCS group versus sham. Treatment comparison *p*-values were considered statistically significant at the two-sided 0.05 level of significance; interaction *p*-values were considered statistically significant at the 0.15 level of significance given the relatively low power for tests of interaction to detect a true interaction. SAS Version 9.4 was used to carry out all analyses.

### Meeting presentation

Presented in part at International Stroke Conference, February 20, 2020; Los Angeles, CA and American Neurological Association, October 8, 2020.

## Results

**Figure 2:**
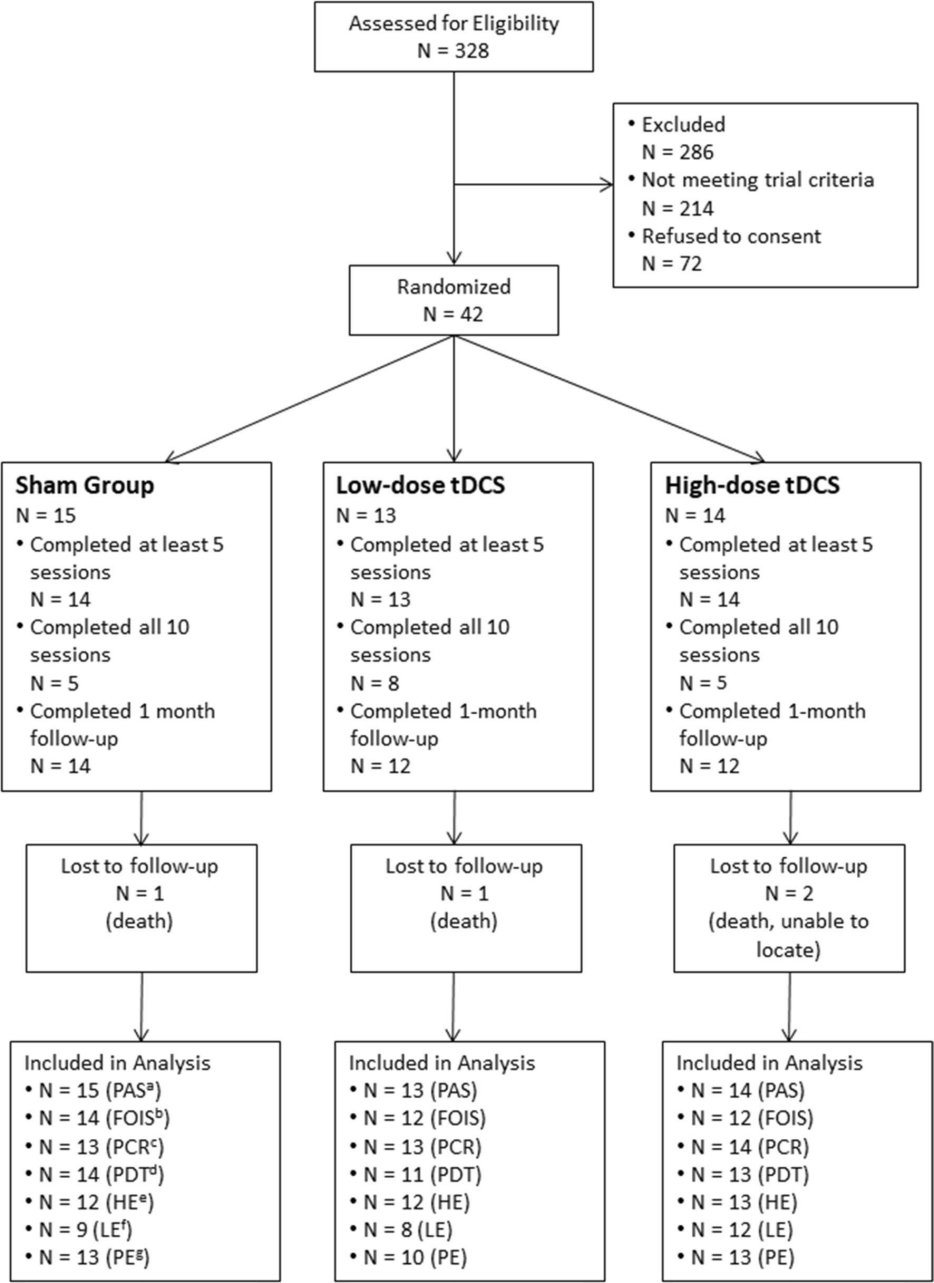
Consort flow diagram showing progress of subjects in the trial Sham and anodal transcranial direct stimulation (atDCS) Groups. (a-Penetration and Aspiration Scale; b-Functional Oral Intake Scale; c-Pharyngeal Constriction Ratio; d- Pharyngeal Delay Time; e-Hyoid Excursion; f-Laryngeal Excursion; g-Pharyngeal Excursion).

A total of 328 subjects were screened for the trial and 42 were enrolled [25 (60%) female, 17 (40%)male; mean age 71 years (SD, 13.2); 34 were of white race (81%) and 5 were black (12%)] (Fig. 2) (Table 1). In comparison the sex, age and race distribution of the screened but non-enrolled cohort were similar and were as follows: mean age 71.58 years (12.69) (*p* = 0.78); male (137; 47.9%), female (149; 52.1%) (*p* = 0.54); 218 (76.2%) white and 48 (16.8%) (*p* = 0.86) The enrolled cohort had moderately severe strokes at study entry (mean NIHSS score 12.3; SD, 5.2). There were 3 deaths (one per arm) and 1 subject was lost to the 30-day follow-up. Overall 18 (43%) subjects completed all 10 sessions and 41 (98%) completed at least 5 stimulation sessions. Forty effortful swallows were completed in 291/ 382 (76%) stimulation sessions. No subject withdrew consent or stopped stimulation due to discomfort; 38 (90%) completed a 30-day follow-up. The mean times in hours (SD) from stroke onset to initiation of experimental intervention was 93.5 (38.8), 101.6 (32.5), 84.1 (34.7), in sham, low-dose and high-dose tDCS groups, respectively (*p* = 0.45).There were no major protocol violations.

**Table 1:** Baseline characteristics of trial cohort in sham and anodal transcranial direct stimulation (atDCS) groups.

Characteristics	Sham N = 15	Low-dose atDCS N = 13	High-dose atDCS N = 14
**Age**			
Mean (SD)	73 (14.1)	72 (13.3)	68 (12.6)
**Sex**			
No. (%) Female	9 (60.0)	5 (38.5)	11 (78.6)
No. (%) Male	6 (40)	8 (61.5)	3 (21.4)
**Race**			
No. (%) White	14 (93.3)	11 (84.6)	9 (64.3)
No. (%) Black	0	2 (15.4)	3 (21.4)
No. (%) Asian	0	0	2 (14.3)
No. (%) Unknown	1 (6.7)	0	0
NIHSS^a^ Score (SD)	11.5 (5.0)	12.5 (6.1)	12.8 (4.7)
PAS^b^ Score (SD)	4 (1.5)	4.1(1.1)	4 (1.2)
FOIS^c^ Score (SD)	2.7 (1.3)	2.8 (1.5)	3.3 (1.6)
**Stroke laterality**			
No. (%) Right Hemisphere	9 (60)	6 (46.2)	3 (21.4)
No. (%) Left Hemisphere	6 (40)	7 (53.8)	11 (78.6)
No. (%) Previous Stroke	2 (13.3)	2 (15.4)	1 (7.1)
No. (%) Hypertension	12 (80)	9 (69.2)	10 (71.4)
No. (%) Diabetes	6 (40)	4 (30.8)	4 (28.6)
No. (%) Atrial Fibrillation	3 (20)	3 (23.1)	7 (50.0)
No. (%) Coronary Artery Disease	2 (13.3)	3 (23.1)	4 (28.6)
**TOAST**^**d**^** Classification (percentage)**			
Cardioembolism	5 (33.3)	6 (46.1)	7 (50)
Large Artery Atherosclerosis	5 (33.3)	3 (23)	3 (21.4)
Undetermined Etiology	4 ((26.6)	3 (23)	3 (21.4)
Other Determined Etiology	1 (6.6)	1 (7.6)	0 (0)
Small Vessel Disease	0 (0)	0 (0)	1 (7.2)
**Timing in Hours- Mean (SD)**			
Time from Onset-to-VFSS^e^	80(38)	91.3 (35.9)	76.1 (32.6)
Time from Onset-to-Enrollment	84.9 (39.6)	98.4 (35.4)	78 (33.1)
Time from Onset-to-Intervention	93.5 (38.8)	101.6 (32.5)	84.1 (34.7)

^a^National Institute of Health Stroke Scale.

^b^Penetration and Aspiration Scale.

^c^Functional Oral Intake Scale.

^d^Trial of Org 10,172 in Acute Stroke Treatment Classification.

^e^Videofluoroscopic Study.

No seizures, deterioration in global neurological, motor or swallowing functions were observed in any arm. Overall, 7 unanticipated serious adverse events occurred that were adjudicated as being unrelated to trial procedures (Table 2).

**Table 2:** Serious adverse events summary.

	Sham (N = 15)		Low-dose tDCS (N = 13)		High-dose tDCS (N = 14)		Overall (N = 42)	
	N	%	N	%	N	%	N	%
At least one serious adverse event	**4**	**27**	**1**	**8**	**2**	**14**	**7**	**17**
	0	0	0	0	0	0	0	0
**Maximum severity experienced**	0	0	0	0	0	0	0	0
Mild	**1**	**7**	0	0	**1**	**7**	**2**	**5**
Moderate	**1**	**7**	0	0	0	0	**1**	**2**
Severe	**1**	**7**	0	0	0	0	**1**	**2**
Life threatening/disabling	0	0	0	0	0	0	0	0
**Fatal**	**1**	**7**	**1**	**8**	**1**	**7**	**3**	**7**
Related to stroke	**1**	**100**	**1**	**100**	**1**	**100**	**3**	**100**
Not Related to stroke	0	0	0	0	0	0	0	0
	0	0	0	0	0	0	0	0
**Maximum relationship to study intervention**	**4**	**27**	**1**	**8**	**2**	**14**	**7**	**17**
Unrelated	**3**	**75**	**1**	**100**	**2**	**100**	**6**	**86**
Unlikely	0	0	0	0	0	0	0	0
Possible	**1**	**25**	0	0	0	0	**1**	**14**
Probable	0	0	0	0	0	0	0	0
Definite	0	0	0	0	0	0	0	0

The relevant parameters for our primary efficacy outcomes are summarized in Table 3. The baseline PAS for all groups combined (n = 42) were approximately normally distributed. No significant differences in change in PAS scores between groups were seen. Adjusting for baseline PAS, NIHSS scores and age, mean [standard error, (SE)] change in PAS scores were − 0.96 (0.33) in sham, − 0.81 (0.36) in Low-Dose and − 0.34 (0.35) in High-Dose; pairwise differences (*p* > 0.40). In the post-hoc analysis adjusting for baseline PRESS score, the mean changes in PAS scores were − 0.97 (0.34) in sham, − 0.83 (0.36) in Low-Dose and − 0.31 (0.36) in High-Dose; pairwise differences (*p* > 0.40). The mean change in PAS scores between subjects completing 10 sessions (n = 18) versus those who did not (n = 24), were − 0.99 (SD 1.44) and − 0.49 (SD 1.41), respectively; after adjustment for age, baseline NIHSS and PAS scores, the least square estimate of the difference between group mean was − 0.57 (SE ± 1.41), *p* = 0.22. The ICC of the raw PAS scores on randomly selected 303 swallow evaluations between the 2 evaluators was 0.67.

**Table 3:** Primary efficacy outcome-intent to treat analysis.

Characteristic	Sham	Low-dose atDCS	High-dose atDCS
**Baseline PAS Score**			
N	15	13	14
Mean (SD)	4 (1.6)	4.1 (1.0)	4.1 (1.1)
Maximum, minimum	2.4, 8.0	1.6, 5.5	2.4, 6.4
**Exit PAS Score**			
N	15	13	14
Mean (SD)	3.2 (1.6)	3.3 (1.4)	3.7 (1.5)
Maximum, minimum	1.0, 5.4	1.2, 7.3	1.2, 6.8
**Change in PAS Score**			
N	15	13	14
Mean (SD)	− 0.8 (1.6)	− 0.8 (1.5)	− 0.4 (1.2)
Maximum, minimum	− 4.3,1.3	− 3.7, 2.5	− 2.2,1.6

Changes in the penetration-aspiration scale (PAS) Scores across sham and anodal transcranial direct stimulation (atDCS) groups.

The relevant parameters for our dietary outcome are outlined in Table 4. There were no significant differences in baseline FOIS scores between groups. After adjusting for baseline FOIS, NIHSS scores and age, mean (SE) change in FOIS scores were 2.07 (0.35) in Sham, 2.46 (0.38) in Low-Dose and 3.05 (0.38) in High-Dose (pairwise *p* > 0.15). A post-hoc analysis of FOIS scores at 30-days showed that 22 /24 subjects in the combined tDCS group had a FOIS score ≥ 5 compared to 8 /14 in sham (*p* = 0.037, Fisher-exact).

**Table 4:** Dietary outcome-intent to treat analysis.

Characteristic	Sham	Low-dose atDCS	High-dose atDCS
**Baseline FOIS Score**			
N	15	13	14
Mean (SD)	2.7 (1.3)	2.8 (1.5)	3.3 (1.6)
Maximum, Minimum	1,6	1,6	1,5
**Exit FOIS Score**			
N	14	12	12
Mean (SD)	4.9 (2.2)	5.4 (0.9)	6.3 (1.0)
Maximum, Minimum	1,7	4,7	4,7
**Change in FOIS Score**			
N	14	12	12
Mean (SD)	2.1 (1.7)	2.5 (1.7)	2.9 (1.2)
Maximum, Minimum	0,5	0,6	1,5

Changes in the functional oral intake scale (FOIS) scores across sham and anodal transcranial direct stimulation (atDCS) groups.

The secondary outcomes for pre-specified swallowing physiological outcomes are summarized in Table 5. No statistically significant difference between intervention groups were seen in any of the pre-defined physiological parameters in adjusted models that included baseline NIHSS score and age. An a priori assessment for effect modification across CBT-LL on the trial intervention showed heterogeneity of intervention effects for the PAS (*p* = 0.116) and FOIS (*p* = 0.017) outcomes (Table 6). In patients below median CBT-LL, the mean change in PAS score was smallest for Sham and largest for High-Dose tDCS, whereas in patients at or above median CBT-LL, the largest mean change in PAS score was seen for Sham. On the contrary, in patients below median CBT-LL, the mean increase in FOIS score was similar across the three groups, whereas in patients at or above median CBT-LL, the mean increase in FOIS score was smallest for Sham and largest for High-Dose tDCS. This suggests that CBT-LL can potentially identify responders versus non-responders using FOIS as an outcome measure.

**Table 5:** Swallowing physiology outcomes across sham and anodal transcranial direct stimulation (atDCS) groups.

Characteristic	Sham	Low-dose atDCS	High-dose atDCS
**Baseline PCR**^**a**^			
N	15	13	14
Mean (SD)	0.12 (0.11)	0.13 (0.15)	0.08 (0.07)
Minimum, maximum	0.02,0.45	0.01,0.54	0.01,0.28
**Change from baseline to day 5 PCR**			
N	13	13	14
Mean (SD)	− 0.02 (0.06)	− 0.1 (0.12)	− 0.01(0.09)
Minimum, maximum	− 0.09,0.09	− 0.29,0.05	− 0.16,0.19
**Baseline PDT**^**b**^			
N	14	11	13
Mean (SD)	13.3 (14.9)	28.2 (29.3)	11.2 (16.1)
Minimum, maximum	0, 42.8	0,88.3	0,60.0
**Change from baseline to day 5 PDT**			
N	14	11	13
Mean (SD)	0.83 (20.6)	− 7.3 (43.4)	5.2 (21.3)
Minimum, maximum	− 37.2,43.3	− 75, 73.3	− 41.7,43.3
**Baseline hyoid excursion**			
N	15	12	13
Mean (SD)	1.08 (0.44)	1.19 (0.31)	1.13 (0.58)
Minimum, maximum	0.39,1.86	0.64,1.65	0.27,2.03
**Change from baseline to day 5 hyoid excursion**			
N	12	12	13
Mean (SD)	0.05 (0.87)	− 0.2 (0.36)	− 0.4 (0.75)
Minimum, maximum	− 1.36,1.46	− 0.66,0.54	− 1.72,1.40
**Baseline laryngeal excursion**			
N	12	9	12
Mean (SD)	1.98 (0.53)	2.07 (1.0)	2.04 (0.77)
Minimum, maximum	1.24,3.13	0.35,3.48	0.75,3.47
**Change from baseline to day 5 laryngeal excursion**			
N	9	8	12
Mean (SD)	0.14 (0.67)	0.17 (1.4)	0.07 (0.81)
Minimum, maximum	− 1.08,1.20	− 1.21,2.61	− 1.12,1.48
**Baseline pharyngeal excursion**			
N	14	10	13
Mean (SD)	1.17 (0.97)	1.22 (0.62)	0.95 (1.12)
Minimum, maximum	− 0.82,3.13	0.11,2.21	− 0.54,3.22
**Change from baseline to day 5 pharyngeal excursion**			
N	13	10	13
Mean (SD)	− 0.07 (0.95)	0.0 (1.02)	0.09 (1.10)
Minimum, maximum	− 1.24,1.64	− 1.46,2.25	− 1.42,1.89

^a^Pharyngeal Constriction Ratio.

^b^Pharyngeal Delay Time.

**Table 6:** Descriptive statistics of penetration-aspiration scale (PAS) and functional oral intake scale (FOIS) with dichotomized corticobulbar tract-lesion load (CBT-LL).

Characteristics	Sham		Low-dose tDCS		High-dose tDCS		
	CBT-LL < Median	CBT-LL ≥ Median	CBT-LL < Median	CBT-LL ≥ Median	CBT-LL < Median	CBT-LL ≥ Median	*p*-value for interaction
Mean (SD) baseline PAS	4.40(1.79)	3.63(1.46)	4.57(0.67)	3.86(1.11)	3.79(0.99)	4.53(1.29)	
Mean (SD) change in PAS	− 0.46 (1.49)	− 1.3 (1.78)	− 0.77 (2.26)	− 0.80 (0.97)	− 0.94 (1.03)	0.13 (1.23)	0.116
Mean (SD) baseline FOIS	2.75(1.39)	2.71(1.38)	2.00(1.22)	3.25(1.58)	3.75(1.58)	2.67(1.51)	
Mean (SD) change in FOIS	3.00(1.83)	1.29(1.25)	3.25(2.06)	2.13(1.46)	2.75(1.04)	3.25(1.50)	0.017

## Discussion

Results from the FEASt trial show that application of atDCS in the acute-subacute stroke phase to the undamaged cerebral hemisphere is safe and well-tolerated within the stimulation parameters used in this investigation. Although the safety of atDCS in AIS patients has been previously reported in small pilot and a recent larger single-center clinical trial^21,39^, the FEASt trial is the first investigation to systematically analyze the safety and tolerability of higher doses of atDCS in this stroke phase.

Our experience validates the feasibility of our approach, where subjects with significant neurological and cognitive impairments in the immediate aftermath of a stroke were able to actively participate in this intervention as a protocol requirement over several sessions. We found a high compliance with the experimental swallowing protocol in this trial. Effortful swallowing maneuvers were successfully elicited in all sessions and most participants were able to perform the pre-specified 40 effortful swallows in 20 min. A significant proportion of subjects did not complete all 10 sessions primarily due to early hospital discharge based on clinical considerations. The mean change in PAS scores in patients completing 10 sessions was higher than those who did not but this difference did not attain statistical significance in an adjusted analysis.

Although swallowing is a multifaceted process, we chose changes in the risk of aspiration as our primary outcome, using an 8-point ordinal PAS scores, as aspiration leads to dietary restrictions, increases the risk of pneumonia and need for tube feeding. Scoring for PAS was done blindly by investigators with expertise in its interpretation though their ICC was sub-optimal and at par with other studies in the field^40,41^. Several factors may have contributed to the variability in scoring. PAS scores assignments are based on multiple considerations including the depth of airway invasion and the patient’s response to it; a recent systematic review shows that misclassification of PAS levels in dysphagia research is common^41,42^. Other investigators have highlighted issues with its construct validity and statistical constraints^40^. Our use of a composite PAS score instead of scoring only on thin consistencies may have also decreased the sensitivity to capture aspiration events. Furthermore, our cohort included stroke subjects with moderate to severe disabilities and their compliance with swallowing instructions may have been additional sources of variability. These considerations place some uncertainty around our results which overall did not show a significant difference between groups for our primary efficacy outcome. This early trial experience highlights some of the limitations of using PAS as an outcome instrument for confirmatory clinical trials in stroke related dysphagia.

We included physiological swallowing outcome measures derived from VFSS since they were anticipated to be more sensitive to changes in swallowing status as well as to elucidate the mechanistic underpinnings for swallowing recovery. These measures included the PCR (a surrogate measure for pharyngeal strength with lower ratio indicating better strength), PDT (a temporal measure for briskness of swallow with lower numbers indicating faster swallow) and HLPE (semi-independent movements that close off the airway and shorten the pharynx)^28–30^. While there are normative data on these metrics in normal and chronic stroke populations, similar data in AIS population is lacking. We saw substantial variability in these measures and some estimates were clearly discordant with the clinical swallowing outcomes. Our experience demonstrates the challenges of using these metrics in AIS trials, and emphasizes the need for developing more robust VFSS protocols, as well as optimization of analytical methods before they can be reliably deployed in multicenter-RCTs.

The overall objective of dysphagia therapies is to safely restore a normal diet. Unlike, PAS, which primarily assesses pharyngeal and laryngeal events during swallowing, changes in diet, as measured by FOIS scores can provide a more holistic assessment of swallowing functions. FOIS is a functional outcome that provides a validated measure of dietary level, is sensitive to changes in swallowing functions and has been tested and validated in stroke population^24^. A theoretical criticism of this scale has been that patients may advance their diet despite persistent swallowing impairment and professional recommendations. We believe this is less likely to have influenced our result. All subjects were randomized and had been clinically stable at their respective dietary levels, the assessors were blinded and computed the scores based on a structured interview. Higher doses of atDCS were associated with higher FOIS scores but group differences were not statistically significant though this comparison may have been underpowered. A post-hoc analysis for minimal clinically important difference (MCID) in dietary intake using a Fisher-exact test (which yields substantially conservative levels of statistical significance), revealed significantly better intake at 30-days in the combined atDCS compared to sham group^43^.

Dysphagic stroke patients usually have more severe deficits and overall fare poorly compared to their non-dysphagic counterparts^1,5,8,9^. We expected that other factors might influence the response to our intervention^44^. We used the CBT-LL variable (derived from an overlay of a lesion map onto a swallowing function relevant canonical probabilistic white matter tract that originated from cortical swallowing centers and traversed the brain to relevant brainstem nuclei) to help create a combined variable of lesion size and relevant lesion location (i.e., the impact of a lesion onto the swallowing-related brain structures). This combined variable overcomes limitations of previous lesion descriptions that relied mostly on lesion volume or the relative components of gray- and white matter in a lesion. We used CBT-LL to better understand the variability in response and found a significant heterogeneity of intervention effect across this variable. These findings are hypothesis generating but suggest that the effects of atDCS are primarily intracortical and its effects on swallowing behavior can be influenced by the extent of damage to the corticobulbar tracts with more damage to one CBT making the contribution and modulation of activity in the other intact hemisphere more important. We anticipate that novel radiological variables such as the CBT-LL may help stratify patients in future studies and assist in predicting responses to experimental interventions in future RCTs.

### Limitations

A significant limitation was accruement of lower than anticipated sample size during the funding period that may have affected the power to detect our efficacy endpoints. Analyses of VFSS based outcomes showed significant variability and our main outcome PAS had suboptimal ICC, which may have undermined our ability to detect a real change. While PAS scores assignments based on VFSS captures the subject’s ability to swallow a limited number of prepared boluses without aspirating it does not translate to true functional swallowing ability in a natural environment. FOIS scores, on the other hand, identifies the patient’s current diet level but does not assess dysphagia related quality of life. Our post-hoc analysis for investigating intervention effects using dichotomized FOIS scores however are susceptible to type I error (false positive); they should therefore be considered hypothesis generating and require further confirmation. Including other outcome measures, such as the Swallowing Quality-of Life could have been a useful adjunct for capturing the subjective perceptions of wellbeing in our trial cohort^45^. Future trials will require a better selection of swallowing parameters as well as further optimization in their acquisition and analysis. It will also need validated metrics to identify minimal clinically important differences (MCID) in meaningful change in swallowing functions. There was suggestion of a dose effect reflected in some of the swallowing measures but not in others. This needs further corroboration and exploration of other doses that may be more effective.

## Conclusion

The results of FEASt demonstrate that application of atDCS to the unaffected hemisphere paired with swallowing exercises in acute stroke patients with post-stroke dysphagia is safe and feasible though it did not decrease the risk of aspiration events in this early phase-2 RCT. It improved dietary intake, which needs further validation in a larger trial. The science behind non-invasive brain stimulation has continued to advance since the inception of this trial. A recent single-center trial demonstrated that 1 mA of daily tDCS significantly improved swallowing function in AIS patients after 4 sessions^39^. A shorter course of in-hospital stimulation, if effective, also carries pragmatic implications, based on observations from FEASt where most subjects were able to complete 5 sessions but adherence declined for more sessions. Meta-analyses from separate studies on motor recovery have suggested a dose–effect relationship although only studies up to 2 mA were included this metanalysis^46^. More recent investigations have revealed that higher dose tDCS (4 mA) is feasible, well tolerated, safe, and leads to stronger activity changes^47,48^. Furthermore, there is growing recognition that improvements in modeling of tDCS induced electrical fields in the human head and utilization of neurophysiological techniques to account for variability in skull conductance can help optimize stimulation parameters for individual subjects^49^.

It is necessary for future trials to consider modifications in stimulation parameters including alternate atDCS doses that incorporate accurate models of electric potential distributions and neurophysiological data, and use of clinically meaningful swallowing outcomes. The heterogeneity of intervention effects across CBT-LL observed in this trial merits further investigation and can be used to stratify subject enrollment and analysis. Our trial experience highlights challenges but also the promise of this technique in improving stroke related dysphagia, which should be investigated further in a confirmatory RCT. A major attraction of this technique is its ease of use, portability, safety profile, and low-cost. It is feasible to use it in multiple settings including at patient’s home using remote monitoring or device settings that allow safe administration. Therefore, if proven effective, this method can have a high penetrance not only in academic, tertiary care settings but can also be scaled up for other outpatient settings.

## Supplementary Information

Supplementary Information 1.

Supplementary Information 2.

Supplementary Information 3.

## Data Availability

All data generated or analysed during this study are included in this published article and its supplementary information files.
