# Supplementary material for: Fostering eating after stroke (FEASt) trial for improving post-stroke dysphagia with non-invasive brain stimulation
Source: Sci Rep. 2022 Jun 10;12:9607. doi: 10.1038/s41598-022-14390-9 (PMC9187742; doi:10.1038/s41598-022-14390-9)
Supplement: Supplementary file 2 — Supplementary Information 2. [file 41598_2022_14390_MOESM2_ESM.docx]

|  | **Sham (N=15)** | | **Low-Dose tDCS (N=13)** | | **High-Dose tDCS (N=14)** | | **Overall (N=42)** | |
| --- | --- | --- | --- | --- | --- | --- | --- | --- |
|  | **N** | **%** | **N** | **%** | **N** | **%** | **N** | **%** |
| At Least One Serious Adverse Event | **4** | **27** | **1** | **8** | **2** | **14** | **7** | **17** |
|  | 0 | 0 | 0 | 0 | 0 | 0 | 0 | 0 |
| Maximum Severity Experienced | 0 | 0 | 0 | 0 | 0 | 0 | 0 | 0 |
| Mild | **1** | **7** | 0 | 0 | **1** | **7** | **2** | **5** |
| Moderate | **1** | **7** | 0 | 0 | 0 | 0 | **1** | **2** |
| Severe | **1** | **7** | 0 | 0 | 0 | 0 | **1** | **2** |
| Life Threatening/Disabling | 0 | 0 | 0 | 0 | 0 | 0 | 0 | 0 |
| Fatal | **1** | **7** | **1** | **8** | **1** | **7** | **3** | **7** |
| Related to Stroke | **1** | **100** | **1** | **100** | **1** | **100** | **3** | **100** |
| Not Related to Stroke | 0 | 0 | 0 | 0 | 0 | 0 | 0 | 0 |
|  | 0 | 0 | 0 | 0 | 0 | 0 | 0 | 0 |
| Maximum Relationship to Study Intervention | **4** | **27** | **1** | **8** | **2** | **14** | **7** | **17** |
| Unrelated | **3** | **75** | **1** | **100** | **2** | **100** | **6** | **86** |
| Unlikely | 0 | 0 | 0 | 0 | 0 | 0 | 0 | 0 |
| Possible | **1** | **25** | 0 | 0 | 0 | 0 | **1** | **14** |
| Probable | 0 | 0 | 0 | 0 | 0 | 0 | 0 | 0 |
| Definite | 0 | 0 | 0 | 0 | 0 | 0 | 0 | 0 |

**Table1.Serious Adverse Events Summary.**

|  | **Sham (N=15)** | | **Low-Dose tDCS(N=13)** | | **High-Dose tDCS (N=14)** | | **Overall (N=42)** | |
| --- | --- | --- | --- | --- | --- | --- | --- | --- |
|  | **N** | **%** | **N** | **%** | **N** | **%** | **N %** | **%** |
| At Least One Adverse Event | **7** | **46** | **3** | **23** | **5** | **35** | **15** | **36** |
|  | 0 | 0 | 0 | 0 | 0 | 0 | 0 | 0 |
| Seizures During Simulation | 0 | 0 | 0 | 0 | 0 | 0 | 0 | 0 |
| Neurological Deterioration | 0 | 0 | 0 | 0 | 0 | 0 | 0 | 0 |
| Limb Deterioration | 0 | 0 | 0 | 0 | 0 | 0 | 0 | 0 |
| Swallowing Deterioration | 0 | 0 | 0 | 0 | 0 | 0 | 0 | 0 |
| Death | **1** | **7** | **1** | **7** | **1** | **7** | **3** | **7** |
| Other | **6** | **40** | **2** | **15** | **4** | **29** | **12** | **29** |
| Cardiac | **2** | **13** | 0 | 0 | 0 | 0 | **2** | **5** |
| Atrial Fibrillation w rapid ventricular rate | **1** | **50** | 0 | 0 | 0 | 0 | **1** | **50** |
| Episodic tachycardia | **1** | **50** | 0 | 0 | 0 | 0 | **1** | **50** |
| Endocrine | 0 | 0 | 0 | 0 | **1** | **7** | **1** | **2** |
| Hyperglycemia | 0 | 0 | 0 | 0 | **1** | **100** | **1** | **100** |
| Gastrointestinal | 0 | 0 | **1** | **8** | 0 | 0 | **1** | **2** |
| Diarrhea | 0 | 0 | **1** | **100** | 0 | 0 | **1** | **100** |
| Infections and Infestations | **1** | **7** | 0 | 0 | **2** | **14** | **3** | **7** |
| Infection | **1** | **100** | 0 | 0 | **2** | **100** | **3** | **100** |
| Metabolism and Nutrition | **1** | **7** | 0 | 0 | 0 | 0 | **1** | **2** |
| Metabolism and nutrition disorders | **1** | **100** | 0 | 0 | 0 | 0 | **1** | **100** |
| Musculoskeletal | 0 | 0 | **1** | **7** | 0 | 0 | **1** | **2** |
| Fall | 0 | 0 | **1** | **100** | 0 | 0 | **1** | **100** |
| Psychiatric | **1** | **7** | 0 | 0 | **1** | **7** | **1** | **2** |
| Depression | **1** | **100** | 0 | 0 | **1** | **100** | **1** | **100** |
| Respiratory | **1** | **7** | **1** | **7** | 0 | 0 | **2** | **5** |
| Pneumonia | 0 | 0 | **1** | **100** | 0 | 0 | **1** | **50** |
| Respiratory distress | **1** | **100** | 0 | 0 | 0 | 0 | **1** | **50** |
| Severe sepsis | 0 | 0 | 0 | 0 | 0 | 0 | 0 | 0 |
| Urinary | 0 | 0 | **1** | **7** | 0 | 0 | **1** | **2** |
| Urinary tract infection | 0 | 0 | **1** | **100** | 0 | 0 | **1** | **100** |
| Vascular | 0 | 0 | 0 | 0 | **2** | **14** | **2** | **5** |
| Deep vein thrombosis | 0 | 0 | 0 | 0 | **1** | **50** | **1** | **50** |
| Pulmonary Embolism | 0 | 0 | 0 | 0 | 0 | 0 | 0 | 0 |
| Thromboembolic event | 0 | 0 | 0 | 0 | **1** | **50** | **1** | **50** |

**Table 2. Summary of All Adverse Events Through Day 5 or End of Stimulation**
