# Supplementary material for: Fostering eating after stroke (FEASt) trial for improving post-stroke dysphagia with non-invasive brain stimulation
Source: Sci Rep. 2022 Jun 10;12:9607. doi: 10.1038/s41598-022-14390-9 (PMC9187742; doi:10.1038/s41598-022-14390-9)
Supplement: Supplementary file 3 — Supplementary Information 3. [file 41598_2022_14390_MOESM3_ESM.docx]

| **The FEASt (Fostering Eating After Stroke with tDCS) Trial** |
| --- |
| Randomized, sham-controlled, double-blinded, single-center study |
| Principal Investigator: Sandeep Kumar, MD  Palmer 127  Beth Israel Deaconess Medical Center Boston, MA 02215 |
| Sponsor: National Institute of Health/National Institute on Deafness and other Communicative Disorders |

LIST OF ABBREVIATIONS

AE Adverse Event

BIDMC Beth Israel Deaconess Medical Center

BUMC Boston University Medical Center

CBT Crticobulbar Tract

DCC Data Coordinating Center

DSMB Data Safety Monitoring Board

FEASt Fostering Eating after Stroke with tDCS

FOIS Functional Oral Intake Scale

HLPE Hyoid, Laryngeal, and Pharyngeal Excursion

Ml Millileter

NIDCD National Institute on Deafness and Other Communication Disorders

NIH National Institutes of Health

NIHSS National Institutes of Health Stroke Scale

PAS Penetration and Aspiration Scale

PCR Pharyngeal Constriction Ratio

PDT Pharyngeal Delay Time

SAE Serious Adverse Event

SLP Speech and Language Pathologist

tDCS Transcranial Direct Current Stimulation

TEAE Treatment-Emergent Adverse Event

TESAE Treatment-Emergent Serious Adverse Event

VFSS Videofluroscopic Swallowing

**Project summary:**

The Fostering Eating After Stroke with tDCS (FEASt) trial is an NIH funded, single-center, phase II-A randomized double-blind trial that will test the safety of tDCS in the acute-subacute phases of stroke recovery and obtain preliminary data on its efficacy in improving swallowing functions in dysphagic stroke patients by combining it concomitantly with swallowing exercises - an approach not previously employed in dysphagia treatment. Subjects will be randomized to one of three intervention arms – a low dose tDCS arm, a high dose tDCS arm, or a sham group. All patient enrollments will occur at Beth Israel Deaconess Medical Center (BIDMC) while the evaluation of videofluoroscopic swallowing studies (VFSS) will be done at Boston University Medical Center (BUMC). A Data Coordination Center at the Boston University School of Public Health (BUSPH) will systematically collect and store all study data. A Data Safety Monitoring Board (DSMB) will review the progress of the study. Figure 1 provides a flowchart of the study overview. The local institutional review boards at BIDMC and BUSPH have approved this trial.

The knowledge gained from this study will help plan a larger confirmatory trial for treating stroke related dysphagia and advance our understanding of important covariates influencing swallowing recovery and response to the proposed intervention.

Figure 1. Trial Overview

**General Information**

Protocol Title: Fostering Eating After Stroke with tDCS (FEASt) trial

Sponsor: National Institute of Health/National Institute on Deafness and other Communicative Disorders

Grant Number: R01DC012584-01A1

Trial Registration: ClinicalTrials.gov (# NCT01919112)

Date of Trial Registration: August 8, 2013

Actual Study Start Date: September 2013

Actual Primary Completion Date: May 31, 2019

Actual Study Completion Date: September 2019

**Rationale and Background Information**

Dysphagia occurs frequently after a stroke and often leads to serious complications such as pneumonia or death ^1-5^ Despite its common occurrence treatment options for stroke related dysphagia remains inadequate. The usual clinical practice revolves around implementing compensatory measures such as dietary modifications, head and neck maneuvers to minimize aspiration, and supervision/assistance during food intake ^6^. Patients with more severe dysphagia usually receive nutritional supplementation via nasogastric or percutaneous gastrostomy tubes till their swallowing improves spontaneously, if at all. The efficacy of these techniques in preventing complications of dysphagia has not been systematically investigated in adequately powered clinical trials though they are accepted as standard of care at most stroke centers. On the other hand, implementation of an intervention that improves swallowing in the early aftermath of a stroke may improve patient outcomes and decrease costs of care. To date, attention has mainly focused on medications, exercise therapy, and different stimulation techniques though their efficacy in stroke patients remains unproven ^7,8^. A recent metaanalysis largely derived from small studies reported some improvement in dysphagia through behavioral interventions and acupuncture, but no effect was found for peripheral or cortical stimulation techniques ^9^.

Non-invasive brain stimulation techniques, such as transcranial direct current stimulation (tDCS) or transcranial magnetic stimulation (TMS) are attractive options for facilitating recovery of swallowing after a stroke. Recovery of swallowing in patients with hemispheric strokes has been demonstrated to occur via compensatory reorganization of the unaffected cerebral hemisphere ^10,11^. The reorganization of the swallowing cortex shows predictable patterns of expansion of the pharyngeal representation in an anterolateral direction, irrespective of lesion site or laterality, making it a potential target for neuromodulating therapies such as tDCS or TMS ^10^. Both, tDCS and TMS are non-invasive brain stimulation techniques but with markedly different characteristics. TMS uses magnetic fields to stimulate brain neurons via an electromagnetic coil held against the skull through which electromagnetic pulses are administered; these pulses are capable of eliciting neuronal action potentials. On the other hand, tDCS uses a constant, low current delivered directly to the area of interest in the brain via small electrodes on the scalp, which either increases (anodal stimulation) or decreases (cathodal stimulation) the neuronal excitability in the targeted brain region, thereby, increasing or decreasing the likelihood of neuronal firing ^12^. The short lived effects of tDCS are mediated by changes in membrane potential while the longer lasting effects occur through synaptic mechanisms similar to induction of neuroplasticity ^13^. tDCS can be combined with physical or behavioral therapies to enhance its effects. In addition, tDCS has a sham mode which makes it possible to examine its effect in a blinded trial paradigm.

tDCS has been shown to improve motor functions in chronic stroke patients ^14-18^ though its effect on improved dysphagia has not been systematically investigated. Furthermore, data about its safety in the acute stroke phase remains sparse. Jefferson and colleagues have recently shown that anodal tDCS can modulate neuronal excitability of the swallowing motor cortex, and make it more amenable to plastic changes ^17^. In a previous pilot study we demonstrated that anodal tDCS application to the unaffected swallowing cortex combined with swallowing exercises was safe and feasible in the acute-subacute stroke phases and showed promise in improving dysphagia ^19^. In light of prior investigations which have demonstrated that the bi-hemispheric innervation of brain stem swallowing centers allow the healthy hemisphere to possess sufficient drive to effect recovery of dysphagia ^10^, we hypothesize that with hemispheric lesions where the brainstem and peripheral structures are intact but the upper circuitry of the swallowing apparatus are dysfunctional, a cortical stimulation technique applied to the healthy swallowing cortex can be effective in restoring swallowing functions.

**Study goals and objectives**

**Primary Aim:** To determine safety and examine effects of 2 different doses of anodal tDCS versus sham stimulation of the swallowing motor cortex of the undamaged hemisphere, combined concomitantly with swallowing exercises, in patients with dysphagia due to an acute-subacute unilateral hemispheric infarction.

Approach:

A) We will collect data on the effect of our intervention by:

i) Analyzing changes in penetration and aspiration between the 2 tDCS and sham groups using by a validated assessment tool, the Penetration and Aspiration Scale (PAS) scores;

ii) Examining effects of differing doses of anodal tDCS versus sham stimulation on several physiological measures of swallowing, derived from videofluoroscopic swallowing studies;

iii) Evaluating durability of any observed effects of tDCS on dietary status as determined by changes in Functional Oral Intake Scale (FOIS) score at study onset and 1 month.

B) We will assess safety by comparing the anticipated and observed incidence of the following major adverse events in each of the 3 groups: seizures, stroke specific mortality, neurological and swallowing deterioration as measured by changes in NIH Stroke Scale (NIHSS), and FOIS and PAS scores, respectively.

**Secondary Aim:** To investigate the impact of subject-specific predictors of dysphagia recovery on the outcome of our proposed intervention.

Approach: We will examine differences in the effect size of our intervention across different strata of subject specific-predictors extracted from our pilot model as well as corticobulbar tract (CBT)-lesion load (as a surrogate measure of lesion size and location) by performing a subgroup analysis using regression models.

**Study design**

The Fostering Eating After Stroke with tDCS (FEASt) trial is an NIH funded, single-center, phase II-A randomized double-blind trial that will test the safety of tDCS in the acute-subacute phases of stroke recovery and obtain preliminary data on its efficacy in improving swallowing functions in dysphagic stroke patients by combining it concomitantly with swallowing exercises - an approach not previously employed in dysphagia treatment. The participant enrollment criteria are as follows:

**Inclusion/exclusion criteria**

INCLUSION CRITERIA

- 21 – 90 years of age
- Between 25 hours (day 2) to 144 (day 6) hours since stroke onset
- Unilateral hemispheric (cortical or subcortical) infarction documented by imaging
- Moderate to severe dysphagia with a PAS score ≥ 4

EXCLUSION CRITERIA

- Prior history of swallowing difficulties
- Any other condition that may independently cause dysphagia
- NIHSS score at enrollment > 25
- Drowsiness or marked cognitive impairment that interferes with participation in swallowing maneuvers
- Severe language comprehension difficulties, requiring only proxy consent for participation
- Intubation ≥ 4 days
- Ongoing use of the following CNS-active medications that can interfere with the effect of tDCS-carbamazepine, pheyntoin, valproic acid and dextromethorphan
- Severe COPD (oxygen dependent)
- Advanced CHF
- Any other medical condition that in the opinion of the investigator significantly shortens life expectancy
- Significant hemorrhagic transformation [defined as dense hematoma >30% of the infarcted area with substantial space-occupying effect or as any hemorrhagic lesion outside the infarcted area] on brain imaging studies prior to enrollment
- Unable to undergo an MRI due to claustrophobia or presence of electrically, magnetically or mechanically activated implant (including cardiac pacemaker), intracerebral vascular clips or any other electrically sensitive support system, metal in any part of the body, including metallic injury to eye, or pregnancy
- Likely candidates for hemicraniectomy, carotid surgery or stenting
- Presence of a potential tDCS risk factor:
  - Damaged skin at site of stimulation
  - Presence of an electrically, magnetically or mechanically activated implant (including cardiac pacemaker), intracerebral vascular clips or any other electrically sensitive support system
  - Metal in any part of the body, including metallic injury to eye
  - Pregnancy - Females of child bearing age will have to undertake a pregnancy test to confirm eligibility, prior to participation
  - History of seizures or unexplained episodes of loss of consciousness

**Detailed methodology**

The trial will prospectively enroll 99 stroke patients with dysphagia due to an acute unilateral hemispheric infarction into the study.

Pre-Screening and Enrollment

All potential study candidates will be pre-screened using the online medical records as well as the patient charts. Patients who fail the bedside swallow evaluation will undergo a standardized VFSS conducted by the Speech and Language Pathologists (SLPs) at BIDMC. Patients who have a PAS score ≥ 4 will be enrolled in the study if they fulfill all other study criteria and provide consent.

Swallowing evaluation

All participants will undergo 3 bedside swallowing evaluations and 2 standardized VFSS to quantify dysphagia severity. The first bedside swallow evaluation will be performed before the first stimulation session, the second will be done after the 5th session and the final evaluation will be done after the final session. The VFSS will be done prior to the first and after the final stimulation session. It will use a total of 3 bolus types and will assess 5 successful swallows. The order of the bolus presentation will not be randomized but given in an incremental way due to the uncertainty about patient’s swallowing status and for safety: 5 ml nectar thick liquid followed by 5 ml pudding, followed by 5 ml thin liquid, followed by 10 ml thin liquid and lastly by 30 ml thin liquid.

If a patient cannot take a bolus because of prior failures, a score of 8 will be automatically assigned to that bolus. The arithmetic mean of the PAS score will be computed for each patient, based on the number of swallows on lateral view. The PCR, PDT, and HLPE will be measured on each successful swallow. Inter-rater reliability of study analyses will be conducted for 30% of the studies, selected randomly. The SLPs will incorporate the information from bedside and VFSS to assign the first (prior to stimulation) and final FOIS score (after stimulation) though the intermediate FOIS score (after 5th session) will only be based on the bedside swallow evaluation.

Randomization

The DCC will institute computer-based randomization to randomize subjects to the two interventions or sham groups by stratifying them according to their baseline PAS score (4-6 vs. 7-8). Two investigators, not involved with stimulation sessions or assessing study outcomes device will receive the randomization code electronically and set the device to active or sham accordingly. For each treatment they will enter the subject ID and setting on a case report form (‘Blinded Information’ form, for the Data Center only).

Intervention

The experimental interventions will be performed over 5 consecutive days for each subject, twice daily (total of 10 sessions). Each session will be of 20 minutes duration and involve anodal tDCS or sham carried simultaneously with swallowing exercises. The subject and all investigators except those involved in programming the device will be blinded to the group allocation; the investigators programming the device will not be involved in any patient evaluation nor delivering the intervention. Subjects will be unable to subjectively distinguish between tDCS versus sham stimulation ^20^.

tDCS

TDCS will be delivered through a battery-driven, constant current stimulator (NeuroConn-DC Stimulator Plus) with the following electrode dimensions: anode (active electrode) will be 3 x 5 cm and the reference electrode will be 5 x7 cm. For real tDCS an anodal current of 2 mA will be delivered for 20 minutes continuously. For sham simulation, the electrodes will be set in exactly the same way as for real tDCS, the current however will only be ramped up for 8 seconds to 2 mA and after 40s gradually decreased to produce a sensation of transient tingling, which is indistinguishable from active stimulation ^20^. The programmers of the device will verify after each session that the device delivered the appropriate stimulation and record their findings.

The high dose group will receive active stimulation during all sessions (i.e. 10 sessions with 2 mA tDCS) whereas the low dose tDCS group will receive active stimulation alternating with sham (i.e. 5 sessions of 2 mA) over 5 days. The sham group will receive sham during all sessions.

We will use the international 10-20 EEG electrode system to guide our electrode montage ^21^. The anode will be placed mid-distance between C3/T3 [left] or C4/T4 [right] over the unaffected hemisphere and the reference electrode over the contralateral supraorbital region. We have previously verified this location using MRI markers on high resolution anatomical brain MRI scans and fMRI scans in healthy patients during swallowing tasks. This montage has been developed with the aim of stimulating the inferolateral regions of the primary sensorimotor and premotor cortex of the unaffected hemisphere, based on studies which consistently point to the importance of the inferolateral primary sensorimotor cortex in regulation of swallowing ^22,23^ as well as information from TMS research that show a pattern of anterolateral expansion of the pharyngeal cortex in the unaffected hemisphere in patients with successful swallowing recovery ^10^.

Swallowing Exercises

All tDCS or sham sessions will be simultaneously combined with swallowing exercises. The exercises will provide the necessary sensory and motor activation of the swallowing cortex and augment the effect of cortical stimulation. We will use the effortful swallow maneuver ^24^ which produces greater cortical activation as compared to dry swallows ^25^.

Patients will be given a lemon flavored lollipop, ice chips, a cold spoon, and/or water spritzer to stimulate saliva production during these sessions. Occurrence of a swallow response will assessed by palpating the excursion of the thyroid cartilage coupled with sound recordings from a laryngeal microphone taped externally to the patient’s throat. Effortful swallows will be alternated with ‘regular’ swallows over the session until the patient has attempted 40 effortful swallows. A total swallowing tally will be kept for each session.

Data acquisition for secondary Aim

The secondary aim constitutes investigating the impact of subject-specific predictors of dysphagia recovery (baseline NIHSS score, dysarthria, CBT-lesion load and intubation) ^26^ on the outcome of our proposed intervention. We will use our trial cohort to extract relevant variables needed for this study arm.

Acquisition and Analysis of MRI Data

To obtain reliable estimates of foci of activation with swallowing tasks and construct coticobulbar tracts, functional MRI and high resolution Diffusion Tensor Imaging (DTI) will be performed on 8 age matched healthy volunteers. The CBT- lesion load will be computed by overlapping the fiber tracts with the lesion maps manually drawn on the MRI scans of the stroke patients enrolled in the trial arm. The functional brain MRI experiments will be conducted on the 8 volunteers recruited for this study. The functional imaging experiment will consist of repeated swallowing trials which will be contrasted with whole hand opening and closing tasks, done at the same frequency as the swallowing tasks. Functional MR images will be acquired with a gradient-echo T2*-weighted MR pulse sequence using our own modification of a sparse temporal sampling method with jittered volume acquisition. Based on our preliminary data, the resulting cortical activation in the ventral motor cortex associated with swallowing will be used as a waypoint mask while the dorsal pontine regions will serve as the seed region in order to reconstruct the corticobulbar fiber tracts.

The CBT-LL variable will be derived by creating a canonical tract of the corticobulbar tract in spatially standardized space using the swallowing related cortical fMRI activation and the posterior pons as seed regions. The DTI scans will be high resolution studies obtained in 12 healthy elderly controls, using a single-shot, spin-echo echoplanar imaging sequence with the following parameters: TR=10 seconds, TE=86.9 ms, resolution 2.6x 2.6 x 2.6 mm3 30 noncollinear diffusion directions with a b value of 1000 s/mm2, and 6 acquisitions with a value of 0 s/mm2. Image acquisition, analysis, construction of corticobulbar tracts and computation of CBT-lesion load will be performed as previously described by Zhu et al in our lab. Overlaying the manually drawn lesion maps derived from DWI sequences of trial participants onto the canonical CBT allowed us to calculate a CBT-lesion-load variable.

**Safety considerations**

A DSMB will be appointed by the Program Officer at the National Institute on Deafness and Other Communication Disorders (NIDCD). Its responsibility will be the oversight of safety of trial participants, review of the safety reports, request of additional data/information (if necessary), and advising the NIDCD regarding the continuation/discontinuation of the study. The Principal Investigator and the Program Officer will also assign an independent Medical Safety Officer (MSO), who will serve as a liaison between the DSMB and the Study team. The MSO can be authorized by the DSMB to break the blind in case of an occurrence of an adverse event or a serious adverse event. However, in an emergency, the Medical Safety Officer can break the blind without prior authorization, if there are serious concerns for the safety of a given study subject. The independent MSO and Principal Investigator will closely monitor the incidence rates of all adverse events reported, whether serious or not, throughout the study and will alert the DSMB if a trend is observed.

The adverse events - seizures, deterioration in global neurological, motor and swallowing functions, and stroke specific mortality during the period of active stimulation will serve as major safety outcome measures. The study DSMB have adopted thresholds for stopping the trial if the incidence of these events exceeds their “natural” probability based on historical data from published literature on acute stroke patients.

I. The incidence of any seizure during the 5 days of active stimulation will be analyzed.

II. Neurological Deterioration will be defined as a ≥ 4-point increase in the total NIHSS score or ≥ 2 points increase in the motor sub-item of the NIHSS score on the same limb between each consecutive day during active or sham stimulation.

III. Swallowing deterioration will be assessed by measuring changes in the FOIS score after the 5th session. To minimize radiation exposure VFSS will not be routinely used for this interim assessment. However, subjects with ≥3 point decrease in the FOIS score will undergo an interim VFSS to document changes in PAS scores. For the purposes of this trial, swallowing deterioration has been defined as an increase in PAS score by ≥ 2 points compared to baseline.

IV. For stroke specific mortality, deaths due to direct consequences of brain injury such as brain edema or seizure will count, but not deaths from recurrent ischemic events, hemorrhage, pneumonia, cardiac events, and infections.

V. Non serious events (headaches, skin erythema, fatigue and visual perceptual changes) will be tabulated to assess patient tolerability. Patient Health Questionnaire-9 (PHQ-9) ^27^ will be used for tracking changes in subject’s mood over the course of the study based on recent reports suggesting an effect of depression ^28^. The PHQ-9 will be administered prior to the first session and after the last session of stimulation, as well as at 1 month.

Evaluation of Safety and Stopping Points

The DSMB will review demographic, baseline, treatment exposure, and safety data, including adverse events, serious adverse events, deteriorations and deaths, which will be summarized prior to each meeting. Data to be reviewed will include tabulations of aggregate data (all data from all patients grouped), tabulations of unblinded comparative outcome data by treatment group, and by-patient listings.

In the role of safety advisory group for this study, the DSMB will:

1. evaluate the incidence of deaths, all adverse events, particularly serious adverse events and those reported with moderate or severe intensity, and other relevant measures of patient safety, patient enrolment and study completion status,
2. determine whether a modification or termination of the study is necessary to ensure patient safety or recommend continuing the study,
3. monitor the conduct of the study, e.g., rate of recruitment, ineligibility, non-compliance with the protocol, dropouts, and balance between treatments with respect to demographics,
4. inspect descriptive statistics of average PAS at baseline and Day 5 and of the change from baseline to Day 5,
5. inspect descriptive statistics of secondary outcomes (PCR, HLPE, PDT, FOIS, NIHSS) and change from baseline in secondary outcomes at each visit in which they are measured,
6. consider whether the trial or dose should be temporarily or permanently stopped based on pre-defined stopping rules for the number of certain type of adverse events. Specifically:
   1. pause/stop enrollment in a dose if 3/10 or 4/20 in that dose exhibit seizures;
   2. pause/stop all enrollment if at least 4/20 or 5/40 patients in both active doses combined experience seizure;
   3. the mortality stopping rule will be the same as the seizure stopping rule; note that the probability for these rules is based on the following assumptions and on the binomial distribution:
      1. we assume the natural history of the condition yields probability of seizure of 6% and probability of mortality of 7%;
      2. probability of stopping the study for safety, if these are the true probabilities, using the above stopping rules, is <5%;
      3. thus if a stopping rule is met, odds are that true rates are higher than 6%/7%;
   4. a swallowing deterioration for an individual patient is defined as follows: A FOIS change from baseline of > 3 points will trigger a repeat, interim swallowing assessment; swallowing deterioration will then be defined as an increase in the PAS score > 2 points compared to baseline score based on this interim assessment. The stopping rule for a swallowing deterioration is:
      1. pause/stop enrollment in a dose if 5/10 or 8/20 in that dose exhibit swallowing deterioration;
      2. Pause/stop all enrollment if at least 8/20 or 13/40 patients in both active doses combined swallowing deterioration; note that the probability for these rules is based on the following assumptions and on the binomial distribution:
         1. we assume the natural history of the condition yields probability of swallowing deterioration of 20%;
         2. probability of stopping the study for safety, if this is the true probability, using the above stopping rules, is <5%;
         3. thus if the stopping rule is met, odds are that true rates are higher than 20%;
   5. a neurological deterioration for an individual patient is defined as a > 4 point worsening from baseline on the NIHSS global impairment score. The same stopping rule for swallowing deterioration will be applied to neurological deterioration.
   6. A limb deterioration for an individual patient is defined as a > 2 point worsening from baseline on the NIHSS motor score for a given limb. The same stopping rule for swallowing deterioration will be applied to limb deterioration.

*These stopping rules will serve as a guideline, not an absolute end-point, for the DSMB to consider stopping the trial.*

All reported AEs and AEs considered by the study investigator to be treatment-related (having a relationship of at least possibly study intervention) will be tabulated for all patients who have received any amount of intervention. Tabulations of serious adverse events will be provided. As part of the AE and serious AE tabulations, the incidence of neurological and swallowing deteriorations will be provided. At regularly scheduled meetings the DSMB will be provided with a copy of all SAE Reports. In addition, the DSMB will be notified of any reportable SAEs as soon as possible in between meetings. Any additional information necessary to evaluate the continued safety of all patients enrolled in this trial will also be provided.

Based on differential mortality rate and the type, severity, and relationship of the AEs to study drug, the DSMB may recommend continuation, suspension, modification of procedures, and/or stopping the trial.

**Follow-up**

Subjects will be followed up by a telephone interview at 1 month after the last stimulation session using a standardized questionnaire to assess their FOIS score. The investigator assessing the subject will be blinded to the intervention allocation.

**Data management and statistical analysis**

Sample Size and Power Calculations

The trial will enroll and randomize 99 subjects; 33 subjects for each of the two active and the sham treatment groups. With the above sample size, after 40% attrition, it is estimated that a difference of 1.0 and 1.15 standard deviations between the active and sham treatment in the mean primary outcome measure of the study can be detected with a type I error rate of 2.5% and power 80% and 90%, respectively. In our pilot study the estimated difference was 1.35 with an approximate standard deviation for the differences in the two groups of 1.2, with a standardized difference of 1.1 standard deviations. Thus we expect to have >87% power to detect the expected differences in our study.

Statistical Analyses

We will conduct Intent-to-Treat (ITT) and Per-Protocol (PP) analyses.

Analytic plan Primary Aim

For the primary analysis – an intent-to-treat approach of all randomized subjects will be used. A linear model will be fitted to the data of the primary outcome variable PAS using PROC MIXED in SAS. The outcome is a change in mean PAS score and treatment will be included as a categorical variable. Additionally baseline PAS and other variables that are identified as confounders will be included as covariates. Adjusted means in the two tDCS groups will be compared to those in the sham group. To control for multiple testing, the Hochberg-Benjamini procedure will be employed, which will result in a positive study result if both null hypotheses are rejected at the 0.05 level or one is rejected at the 0.025 level. Similar analyses will be performed on the FOIS, PCR, HLPE, and PDT outcomes. To assess the durability of the intervention effects, a repeated measures analysis will be used. The outcome will be FOIS score at the onset of the trial and at 1 month. Treatment time and their interactions will be included as categorical variables. Additionally other variables that are identified as confounders will be included as covariates. Adjusted means at the onset of trial and 1 month in the two tDCS groups will be compared to those in the sham group at each time point. Safety analyses will be run on all subjects receiving at least one round of intervention. The incidence rates of adverse events will be described as a whole and by treatment group.

Analytic plan for Secondary Aim

To assess the effect size of the proposed intervention across subject-specific predictors, subgroup analyses will be conducted. For such analyses, we aim to use regression models that include intervention, covariate and covariate by treatment interactions as predictors and PAS scores, PCR, PDT, HLPE as outcomes, to examine the modifying effect of the covariates on the intervention. If a significant interaction is detected, analyses of major efficacy endpoints (PAS scores, PCR, PDT, HLPE) will be performed within each group.

Missing Data

We anticipate a 35% - 40% attrition rate in this study, which includes drop-out, spontaneous recovery and discharge, based on review of our hospital records over the past 5 years. Missing data will be handled by employing multiple imputation methods ^29^.

**Quality assurance**

Interim Monitoring

Once in progress, the study will be monitored by the DSMB, an independent group that will periodically review the results in order to assess safety. No interim efficacy assessment is planned.

Fidelity of stimulation

Two individuals (device programmer) not involved in patient recruitment, stimulation sessions or outcome analysis will be assigned to program the tDCS device. They will receive the randomization code from the DCC and set the device to active or sham accordingly. For each treatment they will enter the subject ID and setting on a case report form (‘Blinded Information’ form, for the Data Center only). The programmer will deliver the device to the treating clinician, who after each treatment will return the device to the programmer. The programmer will verify that the device setting is correct and the device administered the correct dose allocated to the subject to ensure treatment fidelity. This information will be entered into the DCC website.

Training and Standardization

All SLPs involved in performing VFSS have undergone comprehensive training on standardizing the VFSS procedure, which includes uniform methods for bolus presentation, patient positioning, issuing instructions to the patients and obtaining images. They have also been trained to standardize the instructions and recording of the effortful swallowing maneuvers. The performance of all SLPs involved with bedside swallowing exercises will be reviewed randomly and rated to assess their competence in performing and recording the swallowing efforts. SLPs who score 75% or less on the rating scale will need to undergo further training before they can resume working with study subjects. The investigators involved in clinical assessments using NIHSS scores have undergone video training offered by the NINDS and have been certified. The investigators responsible for programming the tDCS device have been trained by representative from the NeuroConn device company on all aspects of device programming, recording and trouble shooting.

Interrater reliability for videos

One-third of all VFSS studies will be randomly selected for inter-rater reliability between two investigators responsible to assigning scores on VFSS.

Fidelity with Swallowing Exercises

Before starting the session, the clinician will attach a laryngeal mic to the patient’s throat (taped externally on the neck) and a second mic to the clinician’s shirt lapel. These microphones are plugged into a multi-input digital recording device. The audio recording device will be used to confirm the occurrence of an effortful swallow. A total tally of the number of swallows will be kept for each session.

Data Quality

The DCC will be responsible for maintaining an up-to-date clinical study database, querying incomplete or inconsistent data. Critical safety data will be cleaned by the DCC, prior to each committee meeting to the extent feasible. The DCC will also is responsible for generation of the statistical tables, data listings, and/or figures included in the DSMB reports.

**Expected outcomes of the study**

Primary Outcome

The trial will use changes in the PAS scores as primary outcome measure to assess efficacy. PAS is a validated 8 point ordinal scale that quantifies penetration and aspiration events observed during VFSS^30^. A cut off PAS score ≥ 4 has been adopted for enrollment as minor degrees of penetration can be seen in normal individuals.

Secondary Outcomes

The secondary outcomes will assess the impact of the intervention on changes in swallowing physiology (a, b & c) and (d) diet.

a) Pharyngeal Constriction Ratio (PCR) is a measure of the pharyngeal area visible in the lateral radiograph view at the point when a bolus is held in the oral cavity divided by the pharyngeal area at the point of maximum pharyngeal constriction during the swallow ^31^.

b) Hyoid, Laryngeal, and Pharyngeal excursion (HLPE) and Pharyngoesophageal (PES) opening will measure the actual excursion of these structures and landmarks from their resting point to maximal excursion ^32^.

c) Pharyngeal Delay Time (PDT) will provide a temporal measure of the briskness of the swallow onset. All the 3 measures (a, b, c) will be collected before the first and after the final session of tDCS/sham.

d) The Functional Oral Intake Scale (FOIS) will be used as a dietary outcome measure. The FOIS has been tested and validated in stroke population and demonstrated to be sensitive to changes in swallowing functions in acute stroke patients ^33^. It will be collected at four time points: 1) prior to initiating tDCS/sham stimulation (same day); 2) after the 5th session of tDCS/sham stimulation; 3) after the last session of tDCS/sham stimulation; and 4) by telephone at 1 month after the last stimulation session using a questionnaire.

**Duration of the project**

The total duration of the clinical trial will be 5 years. The PI can apply for a no-cost extension to the sponsoring agency (NIH/NINDS). The study sponsor may grant study extension based on trial progress. The agency’s decision will be considered final.

**Problems anticipated**

Missing data

Many longitudinal studies have the potential to suffer from problems with missing data. We anticipate a 35% - 40% attrition rate in this study, which includes drop-out, spontaneous recovery and discharge, based on review of our hospital records over the past 5 years.

Dropouts

Any subject who is unable to complete 5 sessions of stimulation/sham will be considered a study drop-out. All the efficacy and safety end-points for these subjects will be analyzed in the Intention-to-Treat analysis but not in a Per-Protocol analysis.

Under-enrollment

This is a single center study and may face trial under-enrollment. The DCC along with the PI, will continuously monitor trial enrollment and report it to the DSMB and the study sponsor at regular intervals.

**Project management**

The PI of the trial is Sandeep Kumar, MD Professor of Neurology at Beth Israel Deaconess Medical Center/Harvard Medical School in Boston. He and his associate work closely together with the speech and language department at BIDMC as well as a team of specialists at Boston University School of Medicine led by Susan Langmore, PhD, CCC-SLP Professor of Otolaryngology. The Principal Investigator is jointly responsible with the DSMB and the IRB for safeguarding the interests of participating subjects and for the conduct of the trials

An independent Data Coordinating Center (DCC) at Boston University will assist the Principal Investigator in providing DSMB members with required documents and data output and distributing meeting minutes. Any communication of DSMB recommendations or conclusions to NIH/NIDCD, if necessary, will be the responsibility of the Principal Investigator

An independent Biostatistician from the DCC not involved in any other aspect of the study will be responsible for producing the statistical reports and analysis necessary for the DSMB.

The independent Medical Safety Officer will determine attribution (relatedness) of unanticipated adverse events to the research protocol, in consultation with the Study Team as needed.

Study Team:

- Sandeep Kumar, MD (PI)

- Sarah Marchina, PhD (Trial coordinator)

- Gottfried Schlaug, MD, PhD (Co-Investigator)

- Susan Langmore, PhD, SLP (Co-Investigator)

- Jessica Pisegna, PhD, SLP (Co-Investigator)

- Vasileios Lioutas, MD (Co-Investigator)

- David Eric Searls, MD (Co-Investigator)

- Joseph Massaro, PhD (Statistician)

- Joseph Palmisano and Na Wang (Data Coordinating Center)

Data Safety Monitoring Board:

- Aneesh Singhal, MD (Chair)

- Sherry Chou, MD, MSc

- Jayme Dowdall, MD

- Jordan Green, PhD, SLP

- Mark Schactman, MHS, MS (Statistician)

Medical Safety Officer

- Shoshana Herzig, MD, MPH (Medical Safety Officer)

NIH Program Officer

- Gordon Hughes, MD

**Ethics**

This trial has been approved by the local institutional review boards at BIDMC and BUSPH. It has been registered with ClinicalTrials.gov (# NCT01919112)

**References:**

1 Gordon, C., Hewer, R. L. & Wade, D. T. Dysphagia in acute stroke. *British medical journal* **295**, 411-414, doi:10.1136/bmj.295.6595.411 (1987).

2 Mann, G., Hankey, G. J. & Cameron, D. Swallowing disorders following acute stroke: prevalence and diagnostic accuracy. *Cerebrovascular diseases* **10**, 380-386, doi:10.1159/000016094 (2000).

3 Odderson, I. R. Acute and subacute stroke rehabilitation. *Archives of physical medicine and rehabilitation* **76**, 889-890, doi:10.1016/s0003-9993(95)80562-1 (1995).

4 Singh, S. & Hamdy, S. Dysphagia in stroke patients. *Postgraduate medical journal* **82**, 383-391, doi:10.1136/pgmj.2005.043281 (2006).

5 Smithard, D. G., O'Neill, P. A., Parks, C. & Morris, J. Complications and outcome after acute stroke. Does dysphagia matter? *Stroke* **27**, 1200-1204, doi:10.1161/01.str.27.7.1200 (1996).

6 Kind, A., Anderson, P., Hind, J., Robbins, J. & Smith, M. Omission of dysphagia therapies in hospital discharge communications. *Dysphagia* **26**, 49-61, doi:10.1007/s00455-009-9266-4 (2011).

7 Bath, P. M., Bath, F. J. & Smithard, D. G. Interventions for dysphagia in acute stroke. *The Cochrane database of systematic reviews*, CD000323, doi:10.1002/14651858.CD000323 (2000).

8 Foley, N., Teasell, R., Salter, K., Kruger, E. & Martino, R. Dysphagia treatment post stroke: a systematic review of randomised controlled trials. *Age and ageing* **37**, 258-264, doi:10.1093/ageing/afn064 (2008).

9 Geeganage, C., Beavan, J., Ellender, S. & Bath, P. M. W. Interventions for dysphagia and nutritional support in acute and subacute stroke. *Cochrane Database of Systematic Reviews* (2012).

10 Hamdy, S. *et al.* Recovery of swallowing after dysphagic stroke relates to functional reorganization in the intact motor cortex. *Gastroenterology* **115**, 1104-1112, doi:10.1016/s0016-5085(98)70081-2 (1998).

11 Li, S. *et al.* Functional magnetic resonance imaging study on dysphagia after unilateral hemispheric stroke: a preliminary study. *Journal of neurology, neurosurgery, and psychiatry* **80**, 1320-1329, doi:10.1136/jnnp.2009.176214 (2009).

12 Nitsche, M. A. *et al.* Transcranial direct current stimulation: State of the art 2008. *Brain stimulation* **1**, 206-223, doi:10.1016/j.brs.2008.06.004 (2008).

13 Schlaug, G., Renga, V. & Nair, D. Transcranial direct current stimulation in stroke recovery. *Archives of neurology* **65**, 1571-1576, doi:10.1001/archneur.65.12.1571 (2008).

14 Baird, A. E. *et al.* Enlargement of human cerebral ischemic lesion volumes measured by diffusion-weighted magnetic resonance imaging. *Annals of neurology* **41**, 581-589, doi:10.1002/ana.410410506 (1997).

15 Hummel, F. *et al.* Effects of non-invasive cortical stimulation on skilled motor function in chronic stroke. *Brain : a journal of neurology* **128**, 490-499, doi:10.1093/brain/awh369 (2005).

16 Iyer, M. B. *et al.* Safety and cognitive effect of frontal DC brain polarization in healthy individuals. *Neurology* **64**, 872-875, doi:10.1212/01.WNL.0000152986.07469.E9 (2005).

17 Jefferson, S., Mistry, S., Singh, S., Rothwell, J. & Hamdy, S. Characterizing the application of transcranial direct current stimulation in human pharyngeal motor cortex. *American journal of physiology. Gastrointestinal and liver physiology* **297**, G1035-1040, doi:10.1152/ajpgi.00294.2009 (2009).

18 Lindenberg, R., Renga, V., Zhu, L. L., Nair, D. & Schlaug, G. Bihemispheric brain stimulation facilitates motor recovery in chronic stroke patients. *Neurology* **75**, 2176-2184, doi:10.1212/WNL.0b013e318202013a (2010).

19 Kumar, S. *et al.* Noninvasive brain stimulation may improve stroke-related dysphagia: a pilot study. *Stroke* **42**, 1035-1040, doi:10.1161/STROKEAHA.110.602128 (2011).

20 Gandiga, P. C., Hummel, F. C. & Cohen, L. G. Transcranial DC stimulation (tDCS): a tool for double-blind sham-controlled clinical studies in brain stimulation. *Clinical neurophysiology : official journal of the International Federation of Clinical Neurophysiology* **117**, 845-850, doi:10.1016/j.clinph.2005.12.003 (2006).

21 Steinmetz, H., Furst, G. & Meyer, B. U. Craniocerebral topography within the international 10-20 system. *Electroencephalography and clinical neurophysiology* **72**, 499-506, doi:10.1016/0013-4694(89)90227-7 (1989).

22 Hamdy, S. *et al.* The cortical topography of human swallowing musculature in health and disease. *Nature medicine* **2**, 1217-1224, doi:10.1038/nm1196-1217 (1996).

23 Ertekin, C. & Aydogdu, I. Neurophysiology of swallowing. *Clinical neurophysiology : official journal of the International Federation of Clinical Neurophysiology* **114**, 2226-2244, doi:10.1016/s1388-2457(03)00237-2 (2003).

24 Lazarus, C., Logemann, J. A., Song, C. W., Rademaker, A. W. & Kahrilas, P. J. Effects of voluntary maneuvers on tongue base function for swallowing. *Folia phoniatrica et logopaedica : official organ of the International Association of Logopedics and Phoniatrics* **54**, 171-176, doi:10.1159/000063192 (2002).

25 Peck, K. K. *et al.* Cortical activation during swallowing rehabilitation maneuvers: a functional MRI study of healthy controls. *The Laryngoscope* **120**, 2153-2159, doi:10.1002/lary.21125 (2010).

26 Kumar, S. *et al.* Recovery of swallowing after dysphagic stroke: an analysis of prognostic factors. *Journal of stroke and cerebrovascular diseases : the official journal of National Stroke Association* **23**, 56-62, doi:10.1016/j.jstrokecerebrovasdis.2012.09.005 (2014).

27 Turner, A. *et al.* Depression screening in stroke: a comparison of alternative measures with the structured diagnostic interview for the diagnostic and statistical manual of mental disorders, fourth edition (major depressive episode) as criterion standard. *Stroke* **43**, 1000-1005, doi:10.1161/STROKEAHA.111.643296 (2012).

28 Kalu, U. G., Sexton, C. E., Loo, C. K. & Ebmeier, K. P. Transcranial direct current stimulation in the treatment of major depression: a meta-analysis. *Psychological medicine* **42**, 1791-1800, doi:10.1017/S0033291711003059 (2012).

29 Rubin, D. B. & Schenker, N. Multiple imputation in health-care databases: an overview and some applications. *Statistics in medicine* **10**, 585-598, doi:10.1002/sim.4780100410 (1991).

30 Rosenbek, J. C., Robbins, J. A., Roecker, E. B., Coyle, J. L. & Wood, J. L. A penetration-aspiration scale. *Dysphagia* **11**, 93-98, doi:10.1007/BF00417897 (1996).

31 Leonard, R. J., Kendall, K. A., McKenzie, S., Goncalves, M. I. & Walker, A. Structural displacements in normal swallowing: a videofluoroscopic study. *Dysphagia* **15**, 146-152, doi:10.1007/s004550010017 (2000).

32 Leonard, R., Belafsky, P. C. & Rees, C. J. Relationship between fluoroscopic and manometric measures of pharyngeal constriction: the pharyngeal constriction ratio. *The Annals of otology, rhinology, and laryngology* **115**, 897-901, doi:10.1177/000348940611501207 (2006).

33 Crary, M. A., Mann, G. D. & Groher, M. E. Initial psychometric assessment of a functional oral intake scale for dysphagia in stroke patients. *Archives of physical medicine and rehabilitation* **86**, 1516-1520, doi:10.1016/j.apmr.2004.11.049 (2005).
